# The Nervous System and Its Functions

**Published:** 1842-07-01

**Authors:** 


THE NERVOUS SYSTEM AND ITS FUNCTIONS. By *Herbert Mayo*, F.R.S. Senior Surgeon of the Middlesex Hospital. London, Parker, pp. 182. 1842.

MR. MAYO informs us, in an advertisement, that " the present volume owes its existence to the following circumstances. I was applied to in the Autumn to prepare some additional letter-press for a second edition of my Engravings of the Brain; and it occurred to me, that it would be suitable to introduce in it a summary account of the functions of the nervous system, which I accordingly drew up, something in the following form. This publication, however, was for the time abandoned; and I likewise have altered my mind about it, and think it better to accompany the plates with a succinct anatomical explanation only, which will be the plan adopted. But in the mean time the form in which I had cast my survey of the nervous system, and the reflections to which it gave rise, appeared to me, if not to have elicited much that is absolutely new, yet to display what had been before discovered with new distinctness and force, and to give to several points, which I had before only half seen, certainty and importance. I therefore again went over the subject carefully, and re-wrote what I now submit to the judgment of my profession, and of the few who take an interest in physiology out of it." So the work is neither new nor old, a rechauffé of what has appeared before with a pinch here and there of something novel or piquant. This releases us from the task of analysis, and compels us to pick out what may seem worth the process.

A chapter of introductory remarks takes up the question of Life and Mind—the separate existence or not of the latter.

Mr. Mayo has handled, and not, we think very happily, this subject before. His argument, so far as it goes, is comprised in the following passage:—

" Then the essential purpose of vegetable existence may be held to be the sustenance of beings endued with consciousness.

For in the lowest animal a glimmering of sensibility is traced; and, as is nature's wont, by many steps of infinite gradation, manifesting the fertility of her resources, and wonderfully combining variety with uniformity, an uninterrupted progression of improving organization, joined to an enlarging scope of sensibility, instinct, and subordinate bodily powers, extends thence upwards to man. And great as may be the final interval between brute intelligence and human reason, yet so much greater is the affinity than the distance, that no disparate modification of organs is required to be made for man; but all that exists in full development in the human brain is found typified, and to a great degree already unfolded in the animals that come nearest to man.

The true purpose and aim of vitality are thus apparent. Life is a force so contrived and used, as to qualify the materials of the inert world for a temporary union with consciousness;—a means how mind may enter into such relations with matter, that it may have its being and part in physical nature, and its faculties developed, and its capabilities and tendencies drawn out and proved (for whatever ulterior purpose) in subjection to and in harmony with her laws.

As we imagine the Supreme Mind to be ubiquitous, infinite, controlling but uncontrolled by matter, so in contrast with these attributes we conceive the finite mind to be bound down to place, and to be dependent on a certain arrangement of matter for its manifestation, each power displayed as the property of a tissue, each agency as the function of an organ.

These views do not lead to materialism. For one cannot disjoin the physiology of the nervous system from mental philosophy, nor investigate the play of its organs without attending to the mind itself. And if equal consideration is given to the two classes of phenomena, it is impossible (so at least it appears to myself,) to avoid the conviction, that they are essentially independent the one of the other, and belong to distinct essences; and that Ipseity, the consciousness of personal being, is not a mode of material existence, nor physical impenetrability an attribute of that which feels and thinks." 7.

Alas! the stiff neck of materialism will not be bent nor broken by this. And, indeed, argument is useless where absolute confutation can touch neither party. Philosophy, in such a case, must bow to Faith, whose assurances are more positive and more consolatory.

### Laws of the Nervous System relating to Sensation and Voluntary Motion.

These Laws form the subject of the first Chapter. The observations of Mr. Mayo are thrown into the form of aphorisms, with a sort of illustrative notes. This saves the trouble of connexion and continuousness, at all events.

*Composition of the Nervous System.*—The nervous system is formed of two dissimilar textures, which are, for the most part, distinguishable by their colour; one being grey, ashen, or cineritious, the other of an opaque white, of the tint termed orange white. The first is principally composed of minute corpuscles. The second, of tubes containing either a gelatinous liquid or a definite filamentous structure. When portions of the nervous system, comprising the two textures, have acquired consistence through maceration in alcohol, the grey is found to have a granular fracture, the white to tear into threads, which are bundles of nerve-tubes.

The distinction of colour obtains in mammalia, birds, reptiles, and in the encephalon of fish. It is distinct, too, in the larger invertebral animals.

*Functions of the Grey and White Matters.*—The grey and white textures have determinably different functions: the white that of transmitting or communicating, the grey that of receiving impressions and originating impulses.

The disposition of the two textures is decisively affirmative of this conclusion. The grey matter forms isolated organs. These are united together by tracts of white matter, the filaments or tubes composing which tend directly towards or from masses of grey matter. Where the nervous system has the most ample organic development, as in the brain, the superficies, or expanded terminal part, consists of the grey texture. It may be added that grey nervous matter is considerably more vascular than white,—a fact of itself indicating a higher function.

*Parts or Segments of the Central Organs of the Nervous System.*—In the simplest animals that possess a distinct nervous system there are two or more central masses of nerves, containing grey matter, and nerves of white matter in connexion with them. The highest animals display a repetition of these parts, with some additions. Thus, says Mr. Mayo:—The central organs of the nervous system in vertebral animals consist of similar parts to those which exist in the invertebrata, and of something more;—of a double cord giving off nerves in pairs to the successive segments of the frame, corresponding with the chain of sensorial nuclei of invertebral animals; and of new organs superimposed upon its anterior, or upper, or cranial extremity. The double cord may be termed the cranio-spinal cord; the succession of parts in it originating pairs of nerves, segments or nuclei; the superimposed organs are the cerebellum and cerebrum.

This repetition of parts and idea of segments is important for the right understanding of the "reflex actions" of the nervous system.

*Conditions necessary for Consciousness*—The spinal cord and medulla oblongata, with the nerves arising from them, are together sufficient to maintain consciousness.

This position is supported by Mr. Lawrence's case of an acephalous fœtus, and by the experiments of Magendie, in which he sliced away the cerebrum and cerebellum until just anterior to the fifth nerve. The animal, so mutilated continues to be as vividly affected by sounds, smells, tastes, punctures of the face, as if it had experienced no further injury than the loss of blood attending the opening of the cranium. If, for example, you pluck a hair of the whisker, or apply a pungent acid to the nose, it tries with its fore-feet to disembarrass itself of the cause of its pain, as if it were unmutilated. Respiration and circulation take place; the movements of the body are not more interfered with, than if the cerebellum alone had been removed. The sensibility of the trunk and limbs has besides suffered no alteration. The animal cries and agitates itself, endeavours to withdraw and to defend itself, when a toe or the sole of the foot is pinched, equally as when the lips or the nose are injured.

Mr. Mayo remarks, that consciousness in these cases depends on the preservation of the part of the medulla oblongata in which the fifth nerves take their origin. He observes that Magendie drew the inference that, in vertebral animals, the perception of all sensations, except those of sight, takes place in the medulla oblongata. "This inference appears to me unsound in two points. It ascribes, by implication, smelling to the fifth nerve, whereas real olfaction belongs to the first. And, which is the important error, it asserts the seat of perception generally to be the medulla oblongata; whereas the facts prove only that sensation does not take place, unless the nerves of the senses and their nuclei of origin preserve continuity with a certain portion of the medulla oblongata."

After remarking that continuity with the segment of the medulla oblongata, in which the fifth nerves rise, is equally necessary to the maintenance of the functions of parts of the nervous system which are situated above or anteriorly to that segment, and that no part of the nervous system severed from that segment participates in consciousness, Mr. Mayo thus argues the matter:—"I dwell upon this proposition to familiarise the reader with the view alone justly deducible from Magendie's experiments above related. The cessation of a function on the separation of a part of the nervous system from this segment, taken alternatively with the suppression of every function on the separate mutilation of that segment alone, does not prove that that segment was the seat of the first. The integrity of the segment of origin of the fifth may be necessary to the preservation of sensibility in the whole trunk, without its being the seat of sensation. The action of the heart in another way may be necessary to secretion, without the heart being the secerning organ.

It is natural perhaps to suspect that the importance of the segment of the medulla oblongata, where the fifth rises, may be here over-rated; and to surmise that its seeming importance results from its central situation, which renders any considerable mutilation of it impracticable without disturbing in a great degree the communication between the anterior and posterior halves of the nervous system, as well as the origins of the seventh and eighth, which have so much to do with the common manifestations of vitality. But the reader may easily get rid of this doubt by observing the difference between a decapitated frog, where the head has been severed at the anterior part of the medulla oblongata, and the same animal when the next minute portion of the medulla is removed: in the first instance, the animal sits collected on its limbs in an attitude prepared for exertion, and if it is hurt, its body and limbs are prompt to consentaneous motion with the full expression of vitality and consciousness. In the second instance, the animal lies at once, extended, relaxed, nerveless, motionless."

The question is an amusing and rather a seductive one in physiological metaphysics, but it is one that will never be settled. It signifies little whether this particular segment of the medulla oblongata be *the* organ of consciousness or not. Consciousness is connected with it so indissolubly, that they cannot be handled apart. Destroy it, you destroy consciousness,—leave it, you leave consciousness, just so perfect or impaired as the nerves of sense are left incomplete or maimed connected with it. A figurative expression of Mr. Mayo's is a good one—it is "the dynamic centre of the nervous system."

Mr. Mayo proceeds:—"Each lateral half of a vertebral animal is separately vitalized. Or the preservation of consciousness in one half is independent of its preservation in the other. Or the continuance of the functions of either lateral half of the cerebro-spinal system depends upon the integrity of the half of the segment of origin of the fifth nerve proper to it.

That is to say, all the modifications of the phenomena of consciousness, which Magendie produced in the preceding experiments upon the whole frame of the vertebral animal, he discovered, may be produced on one side alone, by limiting the mutilation to that side. So destroying the right half of the segment of origin of the fifth, appears to extinguish consciousness on the right side entirely, without in any degree affecting the left. Is it then possible, that by exactly severing in the median plane the two halves of the vitalizing segment, a vertebral animal might be made, temporarily, two separately conscious beings?"

Perhaps Mr. Mayo will try.

*Decussation of the Optic Nerves—Hypothesis of its Object.*—After announcing that the nerves derived from either lateral half of the cranio-spinal cord are distributed to the same side of the frame, and stating that the optic nerves constitute the sole exception to this law, Mr. Mayo goes on to remark, that in all animals with divergent vision there is reason to believe that the optic nerves supply exclusively the opposite eyes. In man, however, for the type of convergent vision, the principle of distribution is different; each optic nerve distributes fasciculi to both eyes. And hereupon Mr. Mayo erects this theory, which, as it is very ingenious, we shall insert.

"Convergent vision is the more perfect function. Now the most sensible spot in the human retina is situated at the optic axis. But this point is situated a little to the outside of the entrance of the optic nerve. Accordingly, it is probable that it is supplied by the fibres which form the outer portion of the optic nerve. But these fasciculi may be traced distinctly backwards, along the outside of the optic tract, to the optic thalamus and corpora geniculata of the same side. The part of the right retina in man, which has the acutest vision, is therefore probably supplied from the right optic tract; the corresponding point in the left retina from the left. And accordingly, so far as the part of the retina situated at the optic axis is concerned, the general law asserted of the distribution of nerves appears to be observed in man in the distribution of the optic.

But if the centre of the right retina, which lies to the right of the entrance of the right optic nerve, is supplied from its right fasciculi, it is probable that the rest of the right portion of the retina is supplied from the same source likewise: and so with the left. And nothing has been ascertained, anatomically, in contravention of this supposition. Then the greater part of either retina in man probably is supplied by the optic nerve of the same side.

But the general direction of this portion of the right retina is towards the left, or towards the left of the common visual axis of both eyes. Now there is a part of the retina of the left eye, which is turned in the same direction. It is, of course, its inner portion. Are we at liberty to surmise, that that portion of the left retina derives its supply of nervous filaments from the inner fasciculi of the left optic nerve, which, at all events, enter the eyeball contiguously to it? If this should be proved to be the case, it would follow that the right optic tract supplies the inner portion of the left retina, as well as the outer portion of the right; for from that tract are derived those decussating fasciculi, which form the inner or left fasciculi of the left optic nerve. Then another, and compensating uniformity, in exchange for that obviously disregarded, would be obtained, and those surfaces of both retinæ, which are directed towards one side, or towards one side of the common visual axis, would prove to be supplied from one optic tract alone.

But, further, it would thus happen that the right optic tract would distribute all its fasciculi to retinal surfaces turned towards one side, and that side the left, or more correctly, the left of the common visual axis. Then suppose a transitional series, in which the same principle should be observed, from animals with perfect convergent vision, to others in which a very small part only of either retina admits of being so employed, it would follow that fewer and fewer of the fasciculi of the right optic tract would continue to go to the right eye. As many only would be so distributed as would supply that lessening portion of the right retina which could be turned towards the left of the common visual axis, the greater portion would cross over to the larger portion of the left retina, now looking towards the left. And finally, when in the completion of the series convergent vision should become wholly lost, the whole of the fasciculi of the right nerve would cross over, maintaining the same principle of uniformity, to be distributed to the left eyeball: the left to the right." 33.

However ingenious this may be it must still be remembered that it is made up of probabilities and surmises, whilst the aim and object of the arrangement would seem, in Mr. Mayo's mind, to be chiefly uniformity. For, he says, "if it (his view) is just, the apparent breach of uniformity is only introduced to make room for a more important observance of the same principle; or to allow of the greatest possible uniformity in the anatomical arrangements for two functions so dissimilar as divergent and convergent vision; and even for the occasional partial introduction of the second more perfect function in the ascending series without any external alteration to betray the sudden step in advance, and the subsequent retreat from it."

*The Reflex Action of the Nervous System.*—Mr. Mayo very ingeniously contrives to *excite* the impression that he is the author of the "reflex" theory, and that he long ago announced the facts it rests upon. He picks out two passages from former publications of his own, and taking them for text, he constructs the hypothesis in question. Poor Dr. Marshall Hall is not burked altogether, but the only evidence of his existence is contained in the following compassionate squeeze.

"Dr. Marshall Hall has given the good name of reflex function to this circle of impression and action, and has added one or two interesting facts in additional illustration of the principle." 50.

How Dr. Hall will like this physiological thimblerig, we will not venture to prophecy. But certainly that gentleman has been strangely dealt with. First of all his views were laughed at or denied. Then, he was told that they were as old as Dr. Whytt. When this was not quite clear, he learnt that he had filched them from Prochaska. Prochaska's writings being rather mystical, Dr. Hall was let alone, and people *did* begin to think that he really had constructed something. But now comes Mr. Mayo, having lain perdu the while, and smirkingly protests that *he's* the man. Indeed Mr. Mayo is one of the greatest of inventors, for Sir Charles Bell and Dr. Hall have both *cabbaged* his discoveries. His contemporaries, we fear, are too envious to do justice to Mr. Mayo's claims. Whether posterity will be more fair, Mr. Mayo, unfortunately, will never learn.

Yet it seems to us that although Mr. Mayo affects to be the father of the "reflex" system, he is somewhat an unnatural one, and is disposed to mangle his own offspring. Perhaps the "Judgment of Solomon" may explain the case.

"In the entire," says Mr. Mayo, "and living frame, the apparatus for sensation is complete in the sentient organ, the sentient nerve, and the segment of the cranio-spinal cord in which the latter rises, taken conjointly.

Vision, for example, is completed in the retina, optic nerve, and optic tubercle.

The following argument appears to me to establish the above proposition.

That the brain is not necessary for sensation in parts supplied with nerves from the medulla oblongata and spinal marrow, is shown by the case of the acephalous infant above cited, and the corresponding experiments of Magendie.

Then two questions remain. Are the proper cerebral organs (the cerebrum and cerebellum) necessary for smell and vision? and is the medulla oblongata necessary for touch and the muscular sense?

That the proper cerebral organs are not necessary for vision, may be inferred from the want of any correspondence of development in fish and birds, between those organs and the optic tubercles and nerves; while the magnitudes of the optic nerves and tubercles are strictly proportionate.

That the medulla oblongata, again, is not the seat of the sensations of the trunk and limbs (as Magendie concluded it to be), may be deduced from the same consideration.

In birds there is much tactile sensibility in the feet, and the muscular sense is required to be well developed in their legs for maintaining their equilibrium. Now in birds the region of the spinal cord which originates the nerves of the inferior extremities is of great size; and the grey matter of the posterior portion which originates the sentient fasciculi, is so large in quantity, as to appear uncovered, forming the floor of a kind of ventricle opened between the posterior columns of white nervous matter. Here then again the sentient organs, the sentient nerves, and their segments of origin, are in size proportionate to each other. But in the medulla oblongata there is no corresponding amplitude." 51.

If we do not altogether misconceive the argument, this runs totally counter to the reflex theory, and indeed to what Mr. Mayo has himself urged a few pages back. The doctrine of the reflex actions implies the absence of sensation (ex necessitate) in the cranio-spinal segment. This argument asserts its presence. It will require much faith to believe that there is really sensation when the hinder limbs of the frog cut in halves jump to the stimulus applied to them—or when the legs of the paraplegiac are agitated on tickling the soles of the feet. There must then, to parody in the grossest way the whimsical expression of Bichât, be an "insensible sensibility," consciousness of which we are unconscious.

The size of the grey matter at the site of origin of nerves of important parts must weigh little against more positive considerations and common experience. The reflex actions in such parts must be varied and considerable, and require a proportionate organization. Besides, we may conceive that volition and sensation, though attributes of another portion of the nervous system, may still, where much exercised, require correspondingly developed instruments in the nervous cords as well as in their points of communication with the central masses. Just as to give effect to the volition of the smith the muscles of his arm and fore-arm must be brawny.

Mr. Mayo, indeed "goes the whole hog," and applies the same mode and extent of reasoning to volition.

"In the entire and living frame, the segments of origin of the motor nerves, with those nerves, are the whole apparatus requisite for originating and transmitting the voluntary impulse.

The argument in proof of this position is essentially the same as in the preceding instance. The size of the segments of origin is proportionate to the size of the voluntary nerves therein arising; while the size of the medulla oblongata and of the cerebral organs observes no such proportion.

This disparity is most conspicuous in some of the larger birds. The size of the medulla oblongata and of the brain is in these totally incommensurate with that of the segments of the spinal cord originating the nerves of the legs.

The difficulty of admitting this view to be correct arises from our habitually conceiving volition to be in the brain, through our confounding the will, used synonymously for intention, determination, resolution, with the voluntary effort to move the limbs. A little reflection will show that there is no reason for adhering to that notion.

The first act in deglutition is certainly voluntary,—that of forcing the food into the pharynx. But the acephalous infant, when food was placed upon its tongue, swallowed it. Why in a perfect infant should more nervous agency be used in swallowing the first morsel than sufficed in the acephalous infant? and why afterwards in an adult is more needed than in an infant?

Then voluntary action is to so great an extent suggested and guided by sensation, that we should naturally look for the place of its origination near the point where sensation is felt. Or let any one recollect how he learnt to skait; how strictly the effort was directed by his sensations, and how slowly each more difficult movement was acquired. At length, indeed, the action became as easy as walking. But walking, running, every complicated voluntary act is, in fact, learnt by the same slow process. Till at last, when we are perfect in each, the conception of going through it is no sooner introduced into the brain and approved and adopted, than with hardly the consciousness of willing it, it is performed. By the facility acquired through use we are led to overlook the interval there was originally between determining upon any corporeal action and its voluntary performance.

When the ostrich hears or sees is pursuer, the instinctive fear and the impulse to flight we may suppose to be in its brain. But the subordinate voluntary efforts, why should they be anywhere but in the great organ which sends off the nerves of its legs? Or let us, allowing the bird more reflection than it probably possesses, suppose it when pursued to deliberate between flight and hiding its head in a bush, the preference of either course, the deliberate determination to run away, for instance, would be a separate mental affection from the consequent voluntary effort to do so. The instinctive impulse towards flight, or the deliberate adoption of it, would be, not the voluntary effort, but its motive." 54.

Too much stress seems to us to be laid by Mr. Mayo on the case of the acephalous infant. It plays the part of Peregrine in "Three and the Deuce," or is like Mrs. Malaprop's Cerberus, three gentlemen at once. Having just proved one thing and made its exit, suddenly we find it on the stage again with a fresh wig and other coloured pantaloons, proving something else. Could this acephalous infant seize the *titty* in its hands, put it in its mouth, and give a deliberate suck, we should say there was evidence of volition. But we take it the feeding was of a different fashion. A spoonful of pap was, in all human probability, emptied into its mouth. The pap descended, for such is its wont, to the fauces and the pharynx. The excited acts and stage of deglutition were produced, and the licking of the mouth (if licking of the mouth there was) had probably very little of the voluntary about it. And besides, in this case, the medulla oblongata was present, and the precise degree of sensation and volition which may stick to that important portion of the nervous system is not satisfactorily determined.

We confess that we have our misgivings on Mr. Mayo's notions of volition. By a singular infelicity he degrades it below instinct, for while he puts the latter in the brain, he places the former in the nerves or in their origins. Volition surely must form a large portion of our intellectual operations—mut be more perfect and less fettered in man than in the lower animals. His brain is developed proportionately—his nerves are not; yet it is in them that Mr. Mayo plants volition. True he says a subordinate volition is in them. We do not understand this. Volition is either absolute or it is not. If it is, there can be no subdivision of it—if it is not, it is no longer volition. Two volitions in the frame, a central and a departmental one, would be very likely to fall out. Indeed we do not see how they should agree. The mental volition would be a kind of abstraction, willing that we should go to bed—while the cranio-spinal volition would be the means of putting on our night cap. So it would come to pass that the cranio-spinal segment would enjoy too opposite properties excited at the same time and in the same way—it would feel and it would not feel—it would will and it would not will, in short it would be an intelligent and a reflex centre. This is more than we can believe.

However it may be, one thing is clear—Dr. Marshall Hall's views and Mr. Mayo's views are as different as day and night. Dr. Hall had given definiteness and distinctness to our notions—Mr. Mayo carries us back again to confusion. For our parts, though at first opposed to Dr. Hall, because, perhaps, we failed to comprehend his reasoning in all its length and breadth, we now prefer his version of the functions of the nervous system.

Mr. Mayo thinks that the office of the anterior pyramids is probably that of transmitting impressions from the brain to the segments of the cranio-spinal cord, not from the cord to the brain. He draws this inference from the fact that the size of the anterior pyramids is not proportionate to that of the spinal cord, but to that of the brain. The other parts of the medulla oblongata have a contrary ratio, and are proportionately likely to contain the upward channels of communication. It will be afterwards seen that the phenomenon of hemiplegia of the opposite side from cerebral lesion, greatly favours the hypothesis above expressed as to the direction of the current of influence in the anterior pyramids.

We conceive that most physiologists have been of this way of thinking, at all events, since the anterior columns of the cord have been appropriated to motion.

"Groups of adjacent segments of the cord are liable to have their functions temporarily or permanently suspended through lesions immediately affecting them, the segments above and below being unaffected.

No cases exemplify this proposition, better, than cases of concussion of the back. Of these, which are very common, most terminate favourably. They may be referred to three classes, in reference to the parts of the cranio-spinal cord which may have been the immediate seat of injury.

1. A violent blow on the middle of the back,—as in the case of a labourer falling through the poles of a scaffolding, and striking the back obliquely against one, his fall being broken, and the principal lesion determined, by this casuality—will cause immediate numbness and paralysis of the legs, in other words, in the parts which derive their supply of nerves from the segments of the cord which have suffered concussion. If the injury of the spinal cord has not exceeded mere concussion, it is usual, by means of appropriate treatment, to obtain sensible daily improvement, and in the course of a few weeks the patient is well, liable only for some time to inflammation of the part that has been thus mechanically disturbed.

2. A parallel injury often happens at the junction of the neck and back. A person pitches head-foremost into a cellar, for example, and the occiput first striking the ground, and obliquely, the head is driven forwards upon the chest, and the strain or mechanical stretching, with the attendant jar or concussion, tells upon the lowest part of the cervical portion of the spinal cord. When the patient comes to himself, he can use neither his arms nor legs. But, in a day or two, feeling and voluntary motion are completely restored in the legs, while, as yet, the arms and hands have experienced no improvement. Then, in favourable cases, in the course of a few weeks, the sensibility and muscular use of the upper extremities likewise come back. In cases of severer injury, the hands and arms never entirely recover, but remain permanently numb and weak, the hands being generally contracted.

It is obvious that the speedy and complete recovery of the legs in these cases, while the nervous power of the upper extremities remains long or permanently impaired, is owing to the essential independence of the lower segments of the cord of the upper. Coupling this class of cases with the preceding, we find in them a complete illustration of the principle, that as long as the vitalizing segment is entire, any of the other segments of the cranio-spinal cord, which remain unimpaired and in sufficient continuity with it, preserve their function.

3. A very interesting case was under my care, resulting from a blow upon the lower and back part of the head, producing concussion of the medulla oblongata. The boy was at first insensible; as he recovered consciousness, his arms and legs were seen to be paralyzed. Improving daily, before long he re-acquired the use of his arms and legs and their feeling was restored; at the same time his intelligence returned, which his looks expressed, but he could not speak, though he evidently made great efforts to do so. A pencil was given to him, when he expressed by writing his feelings and his wants. Gradually, the medulla oblongata recovering from the shock, his speech returned, and he was perfectly restored. It is obvious that the temporary continuance of palsy of the vocal organs, after the use of the limbs had returned, belongs to the same class of phenomena with those of the preceding cases.

In partial disease of a middle portion of the spinal cord, the same phenomena have been observed. The feeling and motion of the legs have continued undisturbed, when the arms have been without motion from contraction or entirely paralyzed.

It has not yet been determined, how much, or what elements of the healthy structure must be left in the intervening diseased or injured segments, to allow of the part of the cord below retaining its functions. I remember an occasion on which it was tried to destroy an ass by pithing it between the atlas and occiput, and the cord appeared to be divided, but the animal went on breathing, and its head continued alive: on closer examination, a small fasciculus, which appeared not thicker than a packing-needle, and had formed the extreme edge of one side of the cord, was found undivided. When it was cut through, the respiratory motions stopped at once.

Disease is still more deceptive. Where there is change of colour and consistence in a part of the brain or spinal cord, pathologists are not entitled to infer that the portion so altered has necessarily lost its functions. In Mr. Stanley's case it was found that a very considerable change in colour and consistence of the posterior half of the spinal cord had existed without sensibly interfering with its known functions. The sensibility which Magendie observed to belong to its superficial fasciculi might, indeed, or might not have been here extinguished. The features of the case do not touch that point.

In cases of paraplegia, there is sometimes a doubt whether the loss of sense and motion in the legs depends upon the interruption of communication with the vitalizing segment of the medulla oblongata, or upon direct affection of the lower part of the spinal marrow. Some light may be thrown upon this doubt by rubbing and pinching the skin of the senseless feet; when, if the segments of the lower portion of the spinal cord are sound, some unconscious action of the muscles of the leg and foot will follow." 62.

All this seems to us a roundabout and clumsy mode of expressing facts that are tolerably familiar. It is commonly imagined, though demonstration be impossible, that each nerve contains filaments that connect it with the spinal segment, and are the medium of the reflex actions, and filaments that connect it with the cerebral centre, and are the medium of consciousness and of volition. The one set may be injured (that is proved) while the other set escapes. If the spinal marrow be cut across, all the sentient and voluntary filaments from the part below the section are cut off; consciousness, therefore, and volition, quoad *that*, are now annihilated. If a partial injury affects the medulla, the consequences will be similarly partial.

Mr. Mayo's allusion to Mr. Stanley's case is a dogmatic argument. The case stands thus. Certain lesions are found to affect sensibly the powers of that portion of the nervous system in which they may have occurred. Certain functions are attributed (not on such evidence as silences all scruples) to the posterior columns of the spinal cord. Those lesions are found in those columns, yet those functions remain. Mr. Stanley says here is a case of difficulty. Mr. Mayo says there is no difficulty at all. "I am satisfied of the function of the part affected, therefore it is clear that such lesions may be unaccompanied with symptoms." This may be true, but it is a dangerous mode of reasoning, and requires a little more caution than Mr. Mayo evinces.

The SECOND CHAPTER is on the

### FUNCTIONS OF THE BRAIN.

Mr. Mayo is a very steady, if not a very fortunate anti-materialist. He has generally an argument against materialism in his pocket, wherever he happens to be. At one time Mr. Mayo could not *conceive* that a thing so immaterial as thought could result from a material combination. But as animals have some thought and much instinct, they too must have an immaterial principle. The immaterial principle must reside in all animated things, down to the very Medusæ.

Mr. Mayo has fitted another string to his bow. "The cerebral organs," says he, "and cranio-spinal cord form, therefore, one system, all the parts of which are united indeed by very elaborate and complicated arrangements, but not in such a manner as to make any one part central. But the mental operations which are manifested in these organs, the seat of many of which has been already shown to be positively determinable, are characterized, not only by their variety and rapidity of combination, but by a principle of unity manifested in their constant reference to the sense of personal individuality, in their subject. And as that singleness of the mind, or personal ipseity, has no correlative in the structure of the brain, the mind, of which it forms so prominent a feature, must be something apart from the brain. And the mind, it may be presumed, is directly and equally connected with the entire nervous system, operating one function in this another in that organ, capable, at its choice, of bending its attention to and of energizing in each singly."

So the immaterial principle pervades the nervous system, is to use Mr. Mayo's own words, "in points and corners of the frame," in our rumps as well as in our heads. The argument runs thus. Personal 'ipseity' is an unity—we cannot *demonstrate* in the brain a spot which shall represent unity—therefore the mind is immaterial. But it would, setting aside other objections, physical and metaphysical, to this reasoning, be hard to affirm that animals have not "singleness of mind or personal ipseity." The goose that cackles on the common, knows her own brood, accompanies her own gander, comes o' nights to her own nest, and punctually attends for her own handful of oats, gives, we take it, sufficient evidence of singleness of mind and personal "ipseity." She never mistakes herself for the goose of a neighbour. She has her own affections, passions, recollections, calculations, just as has the goodman's wife that tends her. Her mind, on a small scale, represents the mind of her mistress. The difference is in degree. The structure of her brain shews, too, no more and no less of a point of unity. The argument, then, must stretch over the animal creation (for *we* know not where it is to stop), and if it proves an immaterial principle for one creature, it proves the same for all. But this is piddling in the sea. It is nonsense, or Pantheism, or both. The attempt to establish the immortality of the soul by physiology or by anatomy is futile. That great point of a Christian's creed has a higher, surer, more consolatory source, than the ward of a hospital, or the bench of a dissecting-room. It is in the Holy Scriptures, or nowhere, that we must look for its establishment.

Mr. Mayo thus explains the relation of the cerebral organs to the cranio-spinal cord. "It is not," he says, "analogous to that between the organs of sense and the cranio-spinal cord, to which it has sometimes been compared. The office of the cranio-spinal cord is not to refer to the organs of sensation for sensible phenomena, to the cerebral organs for reflection and feeling. But the following steps are observed. Impressions made upon the organs of sense are followed by the appropriate sensations being felt in the segments of origin. The office of the cerebral organs is to operate on and elicit more from, or further involve, whatever product the cranio-spinal cord has already obtained as factor to sensible impressions."

We own that to our poor faculties, Mr. Mayo *has* "further involved" the matter. What the precise office of the cerebral organs may be, and what their relation to the cord, is not very clear in Mr. Mayo's hypothesis. It seems to us that he has got into a slough with his theory of the diffusion of mind, and sensation and volition in each spinal segment. The farther he goes, the deeper he'll sink.

Mr. Mayo presents a brief but succinct and clear account of the structure of the cerebrum and cerebellum. We are tempted to extract it.

1. The cerebellum consists externally of what would form, if it admitted of being spread out, a large thin sheet of cineritious matter, which is folded upon a plan not very complicated, the general disposition being in parallel concentric laminæ. Each of the folds, to the smallest, contains white matter, which, collected in the middle of each hemisphere, forms a short column or stem of some thickness. The white matter tears into several orders of filaments, which extend from the superficial grey matter in different directions, establishing the following communications. A great series of filaments has a direction parallel to the foldings of the grey matter, and extends between near and remote laminæ of the same hemisphere. A second series converges from the grey matter of one hemisphere, and issuing at its side and forepart, (the middle peduncle of the cerebellum), crosses in a broad band (the pons Varolii), to coalesce with a corresponding series from the opposite side and bring into communication the two hemispheres. A third, converging from the upper part of one hemisphere, descends within the preceding to form the inferior peduncle, or crus cerebelli, for communicating with the medulla oblongata. A fourth, derived principally from the under and the inner part of the circumference of the hemisphere, converges to form the upper or cerebral peduncle of the cerebellum, and stretches forwards to communicate with the optic thalamus of the same side, and the cerebrum: the grey capsule in the arbor vitæ is filled with white matter from this, the filaments extending to the capsule itself, piercing it, or being replaced on its outside by others which proceed onwards towards the circumference.

2. Having detached the strong band of fasciculi, called the crus cerebelli, to that organ, the rest of the medulla oblongata, consisting inwardly and above of cineritious and white matter mixed, below of white matter alone, stretches towards the cerebrum: the anterior pyramid passing through the pons Varolii, the rest of the medulla crossing over it. The striking feature now presented is the increment of white matter, which springs out of masses of cineritious matter that occur in crowded succession. The pyramid in passing through the pons, divides into coarse fasciculi: the intervals between these and the transverse fasciculi of the pons are filled with grey matter, which originates new white filaments; these reinforce the fasciculi of the pyramid, which issues of many times its former size forming the under and outer layer, or crust, of the crus cerebri. Above, the tubercula quadrigemina furnish a new supply. The mass of ascending filaments, now of some depth, passes through the optic thalamus, which has the form of an egg, pointed forwards, with a deep wedge-shaped part cut out in front, making a space in which the ascending white fasciculi are lodged. The structure of either thalamus is of white and cineritious matter intermingled, tearing in concentric layers disposed around the ascending fasciculi: a vast increment of ascending white filaments is derived from it. The fasciculi, which have now passed through the thalamus, have finally to traverse the corpus striatum, which has a horse-shoe shape open behind, with cross bars or processes uniting the two sides; through these the white fasciculi ascend, having further additions made to them from this source. So various are the sources, and so numerous the fasciculi derived from them, of which the whole series of divergent fasciculi of either hemisphere of the cerebrum is to be constituted.

3. The structure of the cerebrum follows the same type with that of the cerebellum. But the exterior of either hemisphere is formed of a double sheet of cineritious matter, with a fine layer of white interposed between the two. This triple exterior sheet is thrown into folds or convolutions, larger and of a less regular disposition than the laminæ of the cerebellum. The mass of white matter which forms the bulk of each hemisphere, and processes of which fill the hollows of the convolutions, is resolvable into three orders of filaments. First, into filaments placed parallel to the surface, which unite neighbouring convolutions of the same hemisphere, below them others which unite the more remote. Secondly, of fasciculi, which, derived seemingly from the whole cineritious surface of one hemisphere, converge to coalesce with corresponding fasciculi from the opposite. These form two very unequal bands of communication. The upper, or commissura magna, consumes nearly the whole. The anterior commissure unites together the inferior and anterior convolutions of the middle lobes of each hemisphere only. The fornix is partly a commissure of the same description, but principally a means of junction between the optic thalamus and the posterior and middle lobes of the same side. Finally, to unite the hemispheres thus constituted with the parts below, the collected force of ascending filaments derived from the numerous sources recently described diverges in either hemispheres to its entire circumference; every part of this structure is easily unravelled, except where the divergent and convergent fasciculi meet and decussate each other in the hemispheres.

After quoting the experiments of Magendie, of whom he says, that his "talent for experimental physiology transcends that of any other physiologist living or dead"—and of whom we would say that his butcheries have transcended those of all other physiologists put together, while his powers of reasoning justly, on his vivi-sections, are very far from first-rate—Mr. Mayo announces it as "experimentally established that there exist in the enkephalon at least four series of currents or forces, intended to maintain a constant state of equilibrium, and giving rise to sensible disorder whenever that equilibrium is disturbed." Mr. Mayo is a man of more faith than ourselves. We are not so satisfied of the reality of these four currents or forces antagonising one another. It would require something more than the violent mutilations and apocryphal cases hitherto published to prove them.

It is the fashion of some persons to see nothing in the human cerebrum which physically distinguishes it from that of animals lower in the scale than man. If we recollect rightly, Mr. Mayo put forth some such notion in a former work. We criticised it at the time, and are glad to perceive that he has now adopted a more just opinion. " The human encephalon is found," says he, " first to be specially enlarged and amplified in those parts, which are themselves characteristic of the brain of mammalia, namely, the hemispheres of the cerebrum and cerebellum; secondly, to possess these parts of much greater relative size than the animals which come nearest to man, as the dog or the oran-otang; thirdly, to have the same of so great absolute size that they are actually much more voluminous in man than in the horse or cow, which so much exceed man in weight; but, fourthly, to have cerebral organs less in volume than those of the elephant and whale in agreement with the immense disparity of general size,—the brain and cerebellum of the elephant and whale meanwhile not manifesting with their increased size the human proportions of the parts of the enkephalon, but retaining the lower type." This we may safely say—*that*, taking size, complexity, development of those parts which depart most widely from the simplest type, the human cerebrum outstrips all others as Olympus does a mole-hill. This is a plain common-sense view of the matter. The paradoxes of very clever people are often absurd enough.

After noticing that the cerebrum is connected with the apparatus of smell and vision, as well as with the cranio-spinal segments and their nerves, and that the cerebellum has no connexion with those senses, Mr. Mayo gives a guess at the functions of the latter organ. " It may be observed," he remarks, " that the inferior peduncle, or crus cerebelli, on either side attaches itself to the lateral and posterior surface of the medulla oblongata, whereby the fasciculi which descend from it are brought into continuity with those of the spinal cord which contain the posterior or sentient roots of the spinal nerves. Now the class of common sentient cranio-spinal nerves, has as one of its peculiar functions to minister to the inward or bodily sensations. Is it probable that some of the functions of the cerebellum may be to develope instincts connected with that class of sensations?

This idea is consistent with the belief, which so much prevails among phrenologists, that the cerebellum has to do with the sexual impulse. It appears to me, indeed, most probable that the cerebelluum does not originate that impulse. That impulse is a sensational appetite, like hunger, and depends for its existence upon the state of the bodily organs and organs of mere sensation. The argument commonly deduced by phrenologists from pathological phenomena is certainly unsound. As Müller remarks, ' The coincidence of disease of the spinal cord with affection of the genital organs is much more frequent than of disease of the cerebellum." And Cruveilhier even mentions the striking instance of a girl, in whom after death the complete absence of the cerebellum was ascertained, yet who had manifested a strong tendency to a practice growing out of the appetite referred to." We confess that the phrenological argument on the sexual functions of the cerebellum has always seemed to us to be pushed much too far.

Mr. Mayo next enters on the consideration of the intellectual functions of the cerebrum. The following analysis of the admeasurements of Tiedemann proves conclusively, to our minds, that magnitude has, as we might fairly imagine, à priori, a direct and leading influence on the intellectual functions. The *great* facts and large generalizations are those that never mislead. It is individual observations and special circumstances that are so fallacious.

" He (Tiedemann) gives tables of the internal area of the skull in Europeans, Americans, Malays, and Negroes. Of the first he adduces seventy-seven examples; of the second, twenty-four; of the third, thirty-eight; of the fourth, forty-eight. The method of gauging each which he employed was to ascertain the weight of millet-seed required to fill it. I find by averaging his tables, that the European or Caucasian cranium contains forty ounces; the American and Malay, thirty-nine; the Negro cranium thirty-seven only. In other tables in the same paper the length and breadth of the European cranium are shown to exceed those of the Negro cranium. And a very curious fact is added respecting the brain of the Bosjes-woman, who was called the Hottentot Venus. The convolutions of the hemispheres in this specimen [the dimensions of which are likewise of the smallest,] are more strictly symmetrical than is the case in European brains. This feature [absent, I may take occasion to mention, in the brain of a New Zealander which I examined,] is an approach towards the type of the cerebral organization of animals as distinguished from that of human beings. From the oran-otang downwards the two cerebral hemispheres of animals exhibit in their superficial markings great closeness of resemblance; whereas in European brains the resemblance of the gyri of the two sides is general only, and the details of their disposition vary remarkably." 98.

Here we see, in the gross, a great and measureable magnitude of the Caucasian brain, and history demonstrates a corresponding intellectual power. Let those who will try to argue away this remarkable coincidence. To our minds it is worth more than all the vivi-sections that were ever executed.

We remember the time when Mr. Mayo was a staunch antiphrenologist. He has now come round to more moderate and juster notions. Indeed we may observe of this gentleman, as a physiologist, that his mind is naturally too acute to admit of long persistence in erroneous views, although a certain precipitancy and incautiousness oftentimes lead him astray. But hear Mr. Mayo's phrenological creed.

"Are there grounds for adopting the phrenological chart of the cerebral organs?

If there are not sufficient grounds for adopting it, there are, at all events, for examining it. For it cannot be doubted that the vast extent of the layer of cineritious matter, which forms the cortical part of the brain, has some strict physical relation to the conceptions and emotions of the mind; and the probability is very great, that different regions of this stratum are concerned with different classes of conceptions or emotions; and no insurmountable obstacle is apparent to the detection of such relations by measurements even upon the living. And if the primary object of the inquiry should fail, still some new truths will certainly be brought to light in its progress. But there are reasons to hope that the inquiry will prove in the end more or less successful as to its direct object. Accordingly, most of those who have diligently studied the craniological map will be found to believe that its general features, or some of its leading indications, are correct.

The circumstance of a large portion of the cerebral surface being withdrawn from observation, forms the principal difficulty in the inquiry. And there is a circumstance in one of the parts so hidden which necessarily raises some misgivings. The convolutions situated on the inner aspect of the cerebral hemispheres always present a plane superficies. Yet there can be no doubt, that these parts of the cerebral organization must vary in power like the rest; accordingly, *prominence of a part* is but an accident, at all events, in its dynamic development. But if that criterion is lost, what is there to guide one in deciding on a given extent of superficies, which organ is developed, which deficient?

However, these are perhaps not unconquerable difficulties. An intimate acquaintance with the anatomy of the brain, a familiar knowledge of the leading arrangements of its surface, of the uniform principal divisions among the convolutions, and of the varieties in the subdivisions, and a search after their concomitants, if such there are, in the external shape of the skull, may in the end enable an observer to interpret this most obscure page. And one would suppose the obstacles specified must be partly already overcome, when there are those who declare that their reading of the indications of character in the form of the head generally proves correct. Yet one must not build a too confident expectation on such occasional success when it is real. So much is there in physiognomy, and in general extraneous indications of character, to help, without his being aware of it, the guess of the craniologist.

However, for my own part, I profess that I have not sufficiently compared the craniological chart with nature to make my opinion of value as to its correctness. Nevertheless, I have not entirely neglected what opportunities of observation have come in my way. But I certainly am not satisfied that even the principle of distribution of the mental elements which is adopted by phrenologists is just; —that the intellects lie in the front, the moral impulses in the middle, the inferior impulses at the back of the head. Indeed, I have arrived at no more than a few general impressions, rather of a physiognomical than physiological character, which are perhaps hardly worth stating.

I think, *as an artist,* that persons of good capacities and of well-ordered moral impulses, have heads of a certain size or figure, or both.

1. Of the two elements, shape appears to me of more consequence than size.

2. In a small head the forehead should not recede, but should be broad or high; both together give most promise; and the upper part of the head must be round and full, not narrowing towards the vertex, nor the upper part of the sides of the head cut away. Length of head appears immaterial. The late Professor Coleman, whom I had the pleasure of knowing, a man of a social and a most amiable disposition, quick of observation, with considerable reflective powers, of a right heart and a strong head, had a cranium, of which the posterior fifth appeared to have been obliquely sliced off. Shortness, indeed, but without so much abruptness, is almost necessarily a feature in well-formed small heads.

3. If the head is altogether large and ample, a receding forehead does not seem to be at variance with the possession of high intellectual and moral qualities.

4. The head, in addition to being large, very lofty at the middle of the vertex, the forehead sloping, is a mould which has gone with the highest intellect. In this mould was cast the head of Sir Walter Scott. The head of Shakespeare appears to have been similarly shaped.

5. A head of ordinary dimensions, the forehead vertical, but neither high nor broad, the vertex well rounded and of a good height, frequently accompanies great parts. Such was the shape of the heads of Pitt and of Chatham. The forehead of Chatham was something broader than that of his illustrious son.

6. A large eye with long eyelids often accompanies eminent talent; and whether connected with the size of the orbit or with development of the anterior and inferior convolutions of the brain, fullness and squareness of the superciliary region generally go with this feature. In the forehead of Newton, else nowise remarkable, this prominence and largeness of the lower part of the forehead are strikingly pronounced.

7. The head of Napoleon was characterized by its size and squareness, the forehead broad and vertical, and at the same time higher than ordinary: the eyes remarkably large, and the eyelids long. The largeness of the superciliary region was balanced by an equal mass above it.

When large heads are met with in combination with dull capacity, their shape is commonly ungainly, and projections of bone, having no relation to cerebral development, catch the eye.

The worst physical character is great lateral narrowing of the upper part of the head, with a coarse breadth at the lower and middle part. Add to this a mean forehead, and want of symmetry of the two sides, and the portrait is yet deteriorated.

The heads of the ablest and the best, whether large or small, generally look more carefully shaped and better finished than those of commoner persons. The shape, too, in which they are fashioned, seems better filled out; so that the bony boundaries are lost sight of, and the roundness or fulness of the contained organs is the predominant characteristic." 104.

Those who *now* sneer at phrenology in toto, are neither anatomists nor physiologists. That the brain is the *organ* of the mind, whatever the latter may be, is undeniable. That the mind itself is not a simple unity, differing in different individuals in degree only, is as undeniable. A man is born a Byron, a Napoleon, or a Newton, and no circumstances, no mental training could make of the mass of men either of the three. If, then, the mind be a composite thing, built up of various and even clashing qualities, and if the brain be the organ of the mind, the brain must have parts corresponding to those qualities and adapted for their exhibition. Take, if you will, the material hypothesis and it comes to the same point. What reasoning and observation deduce from the manifestations of the brain in its sound state, the phenomena of injuries and disease confirm. The practical difficulty of determining the several mental faculties and their local habitations is great, it may be insurmountable, but the attempt to surmount it is philosophical, and it is by observation only that it can succeed. We do not see, therefore, what there is in phrenology, abstractedly speaking, to laugh at. The truth is that the opposition is that of metaphysics to anatomy and common sense.

Like Mr. Mayo, we will not go the length of allowing the correctness of phrenology in detail. But it is only fair to admit that there may be much truth even in that. When we see, as in the Caucasian race, that size of cranium is the great criterion of intellect—that certain forms of head are historically and by all admission stamped as peculiarly intellectual—that even special mental qualities have a special cranial conformation—when we see all this which common daily observation proves, shall we say that these superficial truths, these facts that swim upon the surface of experience, are all that study, time and reflection can amass—that philosophy must attempt no more without being set in the stocks as a witch, or pelted as a natural? To our apprehension to argue in this way is the fanaticism of prejudice, the confidence of ignorance, the re-enactment of that opposition to induction which has worn so many shapes, and has been foiled in all.

A CHAPTER on the INFLUENCE OF THE NERVOUS SYSTEM OVER THE BODILY FUNCTIONS contains nothing to attract our notice.

Next comes a CHAPTER on PERCEPTION.

In the Chapter on PERCEPTION Mr. Mayo thus dilates on the "Muscular Sense." "The muscular sense immediately feels resistance and motion: and is attended with the idea of force exerted, as an inward sensation; and with distinct conceptions of outness, locality, and direction. The sentient organ of the muscular sense is the voluntary muscles. The nerves, which minister to it, are derived from the ganglionic fasciculi of the fifth cerebral and of the spinal nerves.

The sensations, which suggest to us hardness, softness, weight, lightness, pressure, yielding, support, want of support, are evidently modifications of the feeling of resistance: but they are so mixed up with the impression of something without as their cause, that the terms convey notions of the qualities of bodies rather than the sensations they excite, or bring to our minds objective rather than subjective conceptions.

Sensations of touch often combine with those of the muscular sense. The sensations we have of aëriform, liquid, or solid resistance, for example, are united with and perfected through the tactual feelings excited by these different contacts.

Our notions of elasticity, and of the impenetrability of matter, are ulterior conceptions, founded on the sense of resistance.

Our abstract conception of motion is rendered as if it were sensible to us through this channel.

Our abstract notion of force, in the same way, is realized to the mind through this sense, and is indeed exclusively derived from it. So important are the relations of the muscular sense.

I originally supposed that the ganglionless fasciculi of the fifth and spinal nerves ministered to this sense, being led to form that opinion by observing that the nerves of the eyeball receive but a few minute filaments from the ganglionic fasciculi. And the anatomical fact is still a very puzzling one. Nevertheless, I think its true interpretation is this; that the muscles of the eye do not need a muscular sense: for their exclusive use is to govern the motions of the eyeball; and for that purpose they may be sufficiently instructed by the acute tactual sensibility of the conjunctiva, and by the sense of vision. Then the argument from uniformity will be in favour of the muscular sense belonging to the same class of nerves with the other senses.

But it may be asked, is the sense of resistance in which all the varieties of muscular sensation merge, is this seeming sensation of resistance really *a sensation?* is it indeed more than the mere consciousness of effort? may it not, then, more probably have its seat in the voluntary nerves and their origins, which are the seat of effort? This question may be thus answered. The consciousness of effort is not the same thing with the sense of effort exerted. For example, a person recovering from an attack of palsy has regained the power of moving his foot, but not that of moving his hand. Both efforts he now makes daily with different results. When he tries to move his hand, there is present only the consciousness of the effort. When he tries and succeeds in moving his foot, there is present, besides, the sensation of effort exerted, or of resistance overcome. However, it might be replied, the consciousness of effort, in the first case, is only the consciousness of trying to make an effort: and the argument proves too much, for, in the case advanced, the anterior fasciculi of the nerves are paralyzed as well as the posterior.

A better answer is found in the the phenomena of anæsthesia already adverted to. Considering which phenomena, as well as the remarkable observations of M. Longé and M. Magendie, that the anterior roots of the spinal nerves, and the anterior fasciculi of the spinal marrow even, receive common sensibility through branches of the ganglionic fasciculi, I am disposed to believe that the sensations of different degrees of resistance felt through the muscles must have their communicating medium in the latter class.

In general, the muscular sense is excited by muscular exertion, and goes with it, as its measure. But there are certain sensations which are felt when no muscular exertion is being made, which yet seem to belong to the same source. I allude to the sensations experienced on being lifted, or allowed to sink; for instance, on board ship, when you are lying on the back making no muscular effort, the difference to your sensations is most distinct, produced by the vessel rising or sinking on the water. This sense of support and want of support, what is its seat? I am disposed to think it is still in the muscles. For when we lie perfectly still, we yet have a general sensation of the weight of our body and limbs. I conjecture that feeling to be dependent on the degree of muscular contraction called tone. The sensation of want of support, however, combines further elements.

The sense of resistance gives us, as it has been said, the most definite ideas of motion; we feel and measure each degree of motion by this sense. Now, in the instances just referred to, this sense of motion is obviously disturbed. To the combined muscular sense of resistance and motion, we may then probably attribute the feelings which go with support and want of support.

If the feeling of passive bodily support depends upon this sense, much more so does the feeling of equilibrium, when we stand and walk self-supported.

If we stand or walk in the dark, we rely nearly entirely on the muscular sense to inform us what exertion is necessary to maintain the erect posture; but the sense of touch in our feet contributes a little. In the light, the sense of vision materially aids us. We habitually lean upon our eyesight, in fact, as upon crutches. Accordingly, when a person unaccustomed to do so, looks down a height, and that under circumstances not calculated to disturb his nerves, when he is resting, for instance, against the balustrade round the area at the top of a column, he feels disagreeably the want of visual support, and has an impression that he may topple over. Even the sense of hearing helps us; and its disturbance by unusual noises will increase any existing difficulty in maintaining our equilibrium."

A Scotch metaphysician, in a late number of the Edinburgh Review, has been rather facetious and very angry at the heresy of a muscular sense. According to him we got on well enough with hearing, seeing, tasting, smelling, touching, till anatomy had the impudence to suggest muscular sensation. The metaphysician launches the jargon of his craft on the heads of those who are not content with the five senses. Yet it needs, we think, but a plain man's attention to what occurs in himself to convince him of the reality of the sense in question. We are conscious of the slightest exertion of our muscles—we are conscious of the force with which we exert them, independently of the resistance offered by any foreign body. Let any one try, and he will feel, when he exerts a given muscle, by a voluntary effort, and without any contact of a foreign body with the surface of his own, he will feel, we say, the tremulous action of the fibres of the muscle he exerts. If the muscle be cut or injured there is pain in it. The nerves that supply it contain sensitive filaments. What then does a muscle want that an organ of sensation should have? Indeed, it would be a singular circumstance if that which is the instrument of volition, the constant messenger that the mind employs should be altogether blind and ignorant, and convey no information to that sensorium with which it is in such direct communication. How valueless is a voluntary muscle without feeling is seen in anæsthesia.

It may be urged that the muscular sense is a modification of touch. Suppose that it is so, it is *not* touch, and, if it be not touch, it is distinct from it. It may be true that it usually goes along with touch, but any body may satisfy himself of its independence. Taste is probably a modification of touch too, indeed it is anatomically nearer touch than is muscular sensation. And taste is unquestionably combined with touch in producing the phenomena assigned to it. Yet metaphysics have admitted taste within the pale of the senses. Anatomy, if we are not mistaken, will compel the admission of the muscular sense also.

We do not, ourselves, see why the muscular sense should not depend on the ordinary sensitive filaments of the nerves. The sense in question cannot be separated from the sensation of muscles, and that must depend, if the physiology of the nervous system is not false, on the ganglionic fasciculi. It is going out of the way for a difficulty to suppose otherwise.

It is probable that no change of place or position occurs without the muscular sense participating in the feeling that ensues. But we are not at all satisfied that the sensation of being lifted or sinking must be so exclusively located in the muscles as Mr. Mayo supposes. We have been a good deal upon the sea, but we never found that the vessel could so rise, or fall, or roll as to make us conscious that it was so, without ourselves being so affected that new points of the surfaces were pressed or withdrawn from pressure. Touch and the muscular sense must here go in harness. We do imagine that Mr. Mayo includes in the category of muscular sensations, that sinking at the epigastrium felt when the vessel pitches heavily, or in descending in a swing. This is another matter altogether.

Passing over Mr. Mayo's brief notice of the laws of vision, taste, smell, we arrive at his account of perception, and of the conceptions of time and space and causation. His allusion to Berkeley and to Kant is happy if it is not convincing. "Berkeley," he says, "discerning that we are immediately conscious of sensation alone, was willing to annihilate matter altogether; Boscovich proposed to substitute for it a system of dynamic excitants of sensation; while Kant, more daring still, regarded even space, and time, and causation, as figments of our reason. But to what do the doubts thus raised amount? To the proposition, that our notions of matter, force, motion, and our intuitive conceptions of space and the rest,—being only suggestions of the understanding,—have *possibly* no counterparts out of the understanding. And what is their solution?—That many possibilities exist which contain no portion of probability, and that these are of them.

For, why should Nature have designed to mislead us? Why, when an elaborate mechanism was planned to convey to us ideas of existences beyond our own minds, should that mechanism have been perverted to give us incorrect ideas? Why, when for perception alone so many different avenues of knowledge are opened, should their issues have led each to a different deception, the whole treacherously combining to produce one vast coherent falsehood? Was this easier to nature than speaking truth, or more for our advantage than discerning truth? The magnificent system of the universe, of which Newton unveiled the laws, the constantly increasing wonders of the microscopic world, each so far from us, and with such difficulty brought within the ken of science, why should Nature have framed such remote and august lies? Why not rather believe that her aim has been always to impart truth to us, and that our understandings have the noble power, when carefully trained and diligently exerted, of contemplating her immediate workings, '*veramque patentem Deam?*' "

The impossibility of *refuting* doctrines so opposed to experience as those of Berkeley and Kant is evidence at once of the limitation of our faculties and the greater imperfection of our language. Yet the common sense reply to their bold hypotheses will probably always satisfy the world—*that* although experience be the only basis of our knowledge, and experience *may* be false, they can shew in favour of their opinions but forms of words and suppositions only. On which side the probabilities lie, mankind in general will determine as they have done. Such is our intellectual constitution that *demonstration* of the reality of our conceptions is impossible; the human understanding does not reach so high. But if the *proof* of that reality be not perfect, there is nothing like a shadow of *proof* of their falsehood. If our senses, our only ministers of truth, deceive us, there are at all events no other instruments of knowledge to appeal to. Any hypothesis which excludes their evidence extinguishes all credibility in its own.

We know not a better sample of Mr. Mayo's style than his sketch of the rise and progress of those moral sentiments which happily govern the civilized world. It displays the man of taste and information, and conveys in a few words happily chosen and elegantly disposed the great epochs of our race. We are sure we shall be excused for quoting it.

"In countries by the eastern shores of the Mediterranean civilization began. Between 2000 and 1500 years before the Christian era all its elements were there already put forth. In Egypt science and the arts, though coupled with degrading superstitions; near, for a while within it, the people who preserved the knowledge of the true God; and Phœnicia could boast her commerce and letters, and Asia Minor sent her colonies to Greece and Italy. It was the dawn of the first* day of the world, which with the night that followed lasted 3000 years.

As that day went on, to string exertion to its utmost tension, war, hitherto uninfluential on the general destinies of mankind, broke forth in invasion and conquest; and the Persian, of a middle race unable wholly to emerge from barbarism, overran and subdued Egypt, and Asia Minor, and threatened Greece, whose energies and loftier nature stayed and repelled his myriads.

Then shone the triumphs of that wonderful people; and heroism and valour, the noblest literature, the perfection of imaginative art, the subtlest philosophy, —but not these alone,—the truest conception of morals, formed their chaplet. Let me quote the golden words of the greatest of their sages, to show how admirable was their wisdom.

'God is one, perfect in himself, giving the being and well-being of every creature; what he is I know not, what he is not I know.'—PLAT. *Phæd.*

'That God, not chance, made the world and all creatures, is demonstrable from the reasonable disposition of their parts for use and defence. * * He is such and so great as that he at once sees all, hears all, is everywhere, and orders all.'—XEN. *Mem.*

'The best way of worshipping God is to do what he commands.'—XEN. *Mem.*

'Justice and every other virtue is Wisdom.'—XEN. *Mem.*

'A just man and a happy man are one. He who, in opinion, divided honesty and profit (which are coherent by nature) did an impious act.'

'To be employed is good and beneficial; to be idle hurtful and evil. They that do good are employed; they that spend their time in vain recreations are idle.'—XEN. *Mem.*

'The body, being compounded, is dissolved by death; the soul, being simple, passeth into another life, incapable of corruption.'—PLAT. *Phæd.*

'The souls of the good after death are in a happy estate united to God; the bad suffer condign punishment.'—PLAT. *Phæd.*

'I have not reigned to-day,' if the day had passed that he had not conferred a benefit was the saying of the illustrious pupil of Aristotle, whose military genius rolled back, after two hundred years, the tide of war over Syria, Egypt, (whose strange fate it has been to have fallen the successive prey of every conqueror from Cambyses to Napoleon,—of the Greek, the Roman, the Arab, the Turk,) over Persia and India; and war found then an office which seemed to legitimate it,—the diffusion of civilization; war, now her foe, and fostered by one civilized country alone, which, keeping the front rank in intellectual progress, labours under a strange retardation of moral development.

But, in ruder times, war advanced the progress of mankind; and by war came Roman greatness. Rome, whose virtues were patriotism and courage, her

"* To do justice to the metaphor, it was the third.

The first Milton sang; its night fell with the fall of man. The second closed with that cataclysm, the memory of which haunts the traditions of every nation. The third, long and slow in breaking,

'It seemed that mist of dawning gray
Would never dapple into day;
How heavily it rolled away—
Before the Eastern flame
Rose crimson, and deposed the stars,
And dashed the radiance from their cars,
And filled the earth from his high throne
With lonely lustre, all his own.'

Then the light touched Memnon's statue, and the issuing music chimed the third sunrise."

genius conquest, who made her own the arts and letters of Greece, and the religions of all the world she subjugated; whose function it was to impart civilization, directly or indirectly, to the entire white race of men,—as provincials, as allies, or as the hostile neighbours, who in time were to rend and share her empire. But not civilization alone, but with it true religion, that, where Rome stood, there was left Christendom.

Then fell comparative darkness,—the figurative night of the middle ages,—when rapine and violence lorded it over the world; till nature reclaimed her order, and the arts of peace undermined feudal tower and donjon wall, and the foundations of a higher civilization were cemented.

But in that night of barbarism, there had been the stars, and Alfred had reigned, and justice had sometimes been seen on earth, and the lamp of science and learning had not been extinguished, and there had been watchers for the dawn; and in that night had been the lofty dream of chivalry, which left on the waking world the impress of Honor.

Then began the second day. Towards fifteen centuries of the Christian era, and the same elements reappear in freshened activity, as at the same time before it. For Phœnician adventure, Columbus; for European colonization, that of the New World; for Cadmus, printing; for Moses, Luther.

But what is the promise of this second day? In the first, three words were expounded,—where, when, why. Another interrogative remains—how. Its exposition is already manifest, in the triumphs of physical science, whose great prophet declared, 'Knowledge is power.' But in morals.—Alas! the example of the same eminent person shows that to be wiser is not necessarily to be better, and the experience of centuries has proved how little may result from the soundest ethical views, and from the positive denunciations of religion.

But yet one need not despair;—

One thing certainly contemplated in the scheme of creation is the development of the faculties of mankind, and a progression from barbarism to civilization. Now it is written in our hearts, and declared by revelation, that moral excellence is the noblest attainment. Is nature so powerless for good, or Providence so inconsistent a cause, as to have given man the means of indefinite improvement in that which is secondary and inferior, and to have condemned him to remain stationary and unimproved in that which has the highest worth?

Again, the enlargement of knowledge, and that in a continually-increasing ratio, and its diffusion, are things certain. Is it possible that this cause should fail to make men individually better, by furnishing to many new mental resources and occupation, by habituating increasing numbers to the investigation of truth, and by bringing home to general conviction that temporal prosperity and happiness depend upon self-restraint and moral conduct?

Again, the application of enlarging knowledge to government, the efforts of legislation to prevent one class spreading immorality through another, the increased repression of crime resulting from just views of the object and operation of punishments, the provision of education for every class and wholesome encouragement given to resort to it, and, if, for long to come, the extreme circumference of society must be ground down by iron penury, yet judicious attempts to mitigate that awful allotment, joined above all things with the attainable good of elevating the condition of the class immediately above the lowest, and the influence on every other class resulting from such efforts,—as they are things certain to follow, can they fail to lead to a gradual progression of moral improvement? and may one not add, that, in proportion as men are drawn towards the practice of morality, the influence of religion will be more powerful, which, if it fail to make bad men good, yet never fails to make good men better?

And these our hopes, do they not find encouragement even in the visionary interest which men take in the future progress of their race, and in the allied infirmity of wishing to live in its remembrance?" 158.

These sentiments of Mr. Mayo are noble and just. He is above the sordid bigotry of wishing to restrain the diffusion of knowledge—he contemns the puerile timidity which dreads it—and accepts, as all bold and good men should, the law of God, intellectual as physical, "Let there be light." What arrogance it is, what blasphemy in a privileged class to claim the monopoly of knowledge—to arraign the impartiality of the Deity, and seek to interrupt his dispensations. He has given to the great race that enjoys the dominion of the world, faculties susceptible of improvement and that yearn for it. History has been but the struggle for knowledge and its power. Can those who look back suppose for an instant that it is possible to pause? The battle of mental progress, as of freedom, once begun,
Bequeathed (like it) from sire to son,
Tho' baffled oft, is ever won.

It is the part of wise men to forward and to *direct* it. Nor need we despair of the result. The will of God cannot be evil, nor the destiny he accords his people one to murmur at. We do not fear the moral tendencies of the spread of information. Take the educated classes of Christendom now and compare them with the privileged orders of any former æra. Not only have the coarser vices disappeared, but even those which are tinctured by refinement are softened and subdued. If there is a large and demoralised population in our cities, it is where the benign influence of education has not penetrated, and where the mischievous laws of a dominant class have been festering. But the evil will work its cure. Masses of men cannot be made vicious with impunity, and it will be more costly to feed the poor and to coerce the criminal, than to instruct and give scope to industry. As the world has been marked by great phases of change in the structure of society, so it will continue to be. And it requires no deep penetration to perceive that, as the last six centuries have been distinguished by the rise into power of the middle class, our own time and that to come will be agitated by the heaving of the lower class.

We must conclude. Before we part from Mr. Mayo, we would make one or two remarks. If we have indulged in criticism it is because Mr. Mayo singularly invites it. He puts forward his opinions boldly, and challenges opposition. He cannot complain of receiving what he courts. Nor do we think him fair to others. He can afford to give every man his due, and yet retain a large intellectual capital. For he is a man of unquestionable talent and of liberal attainments. Like Iago, we would say—

Beware, my Lord, of jealousy.

Were Mr. Mayo candidly to admit the claims of others, others would be more free to admit his own.

We are not in the habit of pronouncing judicial opinions on books. It is an assumption of the critic only tolerated, yet not justified, because he is concealed. We shall therefore say no more than this—we would advise Mr. Mayo, in a subsequent edition to reconsider carefully some portions of his work, and while he softens the expression of what is doubtful, give extension and further development to what is known. In the volume as it is, there is much that is excellent, much that proves the high place which its author deservedly holds as a physiologist. We therefore recommend it strongly.

